# Paediatric cataract surgery with 27G vitrectomy instrumentation: the Ghent University Hospital Experience

**DOI:** 10.3389/fmed.2023.1197984

**Published:** 2023-08-03

**Authors:** Hwei Wuen Chan, Filip Van den Broeck, Axelle Cools, Sophie Walraedt, Inge Joniau, Hannah Verdin, Irina Balikova, Stefaan Van Nuffel, Patricia Delbeke, Elfride De Baere, Bart P. Leroy, Fanny Nerinckx

**Affiliations:** ^1^Department of Ophthalmology, National University Hospital, Singapore, Singapore; ^2^Department of Ophthalmology, National University Singapore, Singapore, Singapore; ^3^Department of Head and Skin, Ghent University, Ghent, Belgium; ^4^Department of Ophthalmology, Ghent University Hospital, Ghent, Belgium; ^5^Center for Medical Genetics, Ghent University Hospital, Ghent University, Ghent, Belgium; ^6^Department of Biomolecular Medicine, Ghent University, Ghent, Belgium; ^7^Department of Ophthalmology, University Hospitals Leuven, Leuven, Belgium; ^8^Schilde Eye Centre, Schilde, Belgium; ^9^Department of Ophthalmology, AZ Sint-Jan, Bruges, Belgium; ^10^Division of Ophthalmology, The Children’s Hospital of Philadelphia, Philadelphia, PA, United States

**Keywords:** paediatric cataract, congenital cataract, nystagmus, galactosaemia, torches, 27G vitrectomy

## Abstract

**Objective:**

To describe a cohort of paediatric patients who underwent unilateral or bilateral lens extractions at Ghent University hospital using the Dutch Ophthalmic Research Center (D.O.R.C.) ultra-short 27G vitrectomy system.

**Methods:**

Retrospective analysis of the medical and surgical records of all children that underwent lens extraction between September 2016 and September 2020 using the D.O.R.C. ultra-short 27G vitrectomy system.

**Results:**

Seventy-two eyes of 52 patients were included. The most important aetiologies in this study were of secondary (25.5%), developmental (13.7%), or genetic (13.7%) nature. No definitive cause could be established in more than a quarter of cases (27.5%) despite extensive work-up, them being deemed idiopathic. The remainder of cases (19.6%) was not assigned a final aetiologic designation at the time of the study due to contradicting or missing diagnostic data. This study could not identify any cataract cases related to infection or trauma. Surgical complications rate was 61.1% of which posterior capsule opacification was the most frequent with a rate of 25%. A significant short-term postoperative best-corrected visual acuity gain (≤ −0.2 LogMAR) was observed in 60.5% of eyes for which usable acuity data were available (*n* = 38).

**Conclusion:**

Many different instruments and techniques have been described and used in the context of paediatric lens extractions, each with its advantages and disadvantages. This study illustrates that an ultra-short 27G vitrectomy system can be used to perform paediatric lens extractions with good surgical outcomes. Further studies and comparative trials are needed to ascertain this further.

## 1. Introduction

Paediatric cataract is a treatable, leading cause of visual impairment in children accounting for 5%–20% (approximately 200,000 cases) of childhood blindness worldwide (1). The estimated median global prevalence is 1.03 per 10,000 children (0.32–22.9 per 10,000) with incidence ranging from 1.8 to 3.6 per 10,000 per year (2). Variations from population to population and between developed vs. developing nations exist due to differences in screening programmes, immunisation protocol and population-based genetics (3, 4). Despite being rare, blindness related to paediatric cataract remains a cause of great concern with significant psychological and socioeconomic repercussions when left untreated or when treatment is delayed.

Cataract is defined as an opacification of the crystalline lens and results in reduced visual acuity and contrast sensitivity when significant. In children, this is of particular importance as visual axis obstruction during neurosensory development can lead to irreversible deprivational amblyopia in the absence of timely intervention. Paediatric cataract can be categorised into congenital or acquired, unilateral or bilateral and can present in isolation or as part of a syndrome. Causes of paediatric cataract include genetic (with autosomal dominant transmission accounting for at least 75% of cases) (5), metabolic (namely galactosaemia and galactokinase deficiency), infectious (toxoplasmosis, toxocariasis, congenital rubella syndrome, cytomegalovirus, herpes simplex and syphilis), syndromic/systemic and developmental [such as X-linked oculocerebrorenal syndrome of Lowe, Hallermann-Streiff-François syndrome and persistent foetal vasculature (PFV)], secondary causes (such as chronic retinal detachment, retinopathy of prematurity, intraocular tumour, steroid therapy, uveitis, e.g., juvenile idiopathic arthritis), traumatic and idiopathic. Most paediatric cataracts are treatable and should not be ignored. Optimal management of paediatric cataract includes early identification, prompt referral (within days or weeks) and a multidisciplinary care approach comprising experienced clinicians, orthoptists, and optometrists. Indications for cataract surgery include visually significant central cataracts of >3 mm in diameter, dense nuclear cataracts, cataracts obstructing fundus view, cataracts associated with strabismus and nystagmus (6, 7). Visually significant cataract requires timely surgical intervention and must be complemented by comprehensive pre-operative evaluation and post-operative amblyopia management. Pre-operative evaluation, depending on the presenting features, usually includes at minimum a complete workup by the paediatrician to exclude developmental, infective, metabolic, or genetic aetiology, meticulous ophthalmic examination, and pre-anaesthesia assessment. Timing of surgery is critical, particularly in infants where rapid visual decline ensues beyond the first 2 months of life (4). Management of infantile congenital cataract is challenging due to the delicate balance between early intervention (risks associated with general anaesthesia and secondary glaucoma) and delayed intervention resulting in irreversible visual impairment.

In young children, routine paediatric cataract surgery might require examination under anaesthesia (EUA) combined with intraoperative intraocular pressure and corneal diameter measurement, keratometry, biometry and B-scan ultrasonography (where applicable). A typical approach to lens extraction in children involves the following steps: anterior capsulotomy with capsulorhexis forceps, hydrodelamination and lens aspiration using an irrigation/aspiration (I/A) probe or a vitrectomy cutter (20-23G depending on cataract density), posterior capsulotomy and anterior vitrectomy with or without intraocular lens (IOL) insertion depending on the age. IOL implantation is usually reserved for children above the age of 2 when the eye has achieved 80% of its growth. In a healthy developing eye, the rate of axial length increase is greatest up until 2 years of age, especially between age 1–2 years (8–10). Unlike adults, young children have poor scleral rigidity with a tendency for the anterior chamber to collapse during surgery. Strategies to circumvent this include smaller watertight corneal incisions and the use of viscoelastic. Technological advances in surgical instrumentation (from 20G to 27G vitrectomy cutter) and enhanced fluidics aid in the refinement of surgical techniques allowing for safer and effective surgery. Nevertheless, paediatric cataract surgery, especially infantile cases, should be performed by experienced paediatric cataract surgeons within a team setting with expert paediatric anaesthesia, postoperative paediatric medical and nursing support (4, 11).

This study describes the experience using the customised D.O.R.C. ultra-short 27G vitrectomy system in paediatric cataract surgery at the Ghent University Hospital (GUH; Ghent, Belgium). We report the pre-operative clinical characteristics and clinical outcomes of the Ghent paediatric cataract cohort.

## 2. Materials and methods

This is a retrospective study of paediatric patients who underwent cataract extraction at the GUH between September 2016 and September 2020. Data of 72 eyes of 52 patients were extracted from the GUH database.

Data collected include birth history, medical history, family history, best-corrected visual acuity (BCVA), cataract morphology, metabolic and infection screen, and genetic analysis. Pre-operative data obtained under general anaesthesia included: cataract morphology, autorefraction, B-scan ultrasonography, rebound tonometry (iCare, Tiolat Oy, Helsinki, Finland), keratometry, axial length measurement using A-scan ultrasonography. Surgical duration was captured from ‘knife-to-skin’ to ‘skin closure’. Post-operative data included BCVA, refraction and recorded complications related to the surgery.

Visual acuity assessment for the various age groups: preverbal children up to 12 months, 12–24 months, 2–4 years of age was performed using Teller acuity cards (TAC), Cardiff cards and Kay pictures, respectively. These measurements were converted to LogMAR for statistical analyses. In newborns, visual acuity was assessed by observing their visual behaviour to different stimuli. For all other patients, BCVA was recorded in LogMAR (and converted to this if given in Snellen for statistical analysis). Visual acuity of 1/60 or counting fingers, hand movements, light perception and no perception of light was recorded as 1.98, 2.28, 2.6, and 3.0 LogMAR, respectively.

Genetic testing was performed in the clinical setting at the Centre for Medical Genetics Ghent, GUH, using the cataract targeted clinical exome panel comprising 65 genes *ABHD12*, *ADAMTSL4*, *AGK*, *BCOR*, *BEST1*, *BFSP1*, *BFSP2*, *CHMP4B*, *COL18A1*, *COL2A1*, *CRYAA*, *CRYAB*, *CRYBA1*, *CRYBA2*, *CRYBA4*, *CRYBB1*, *CRYBB2*, *CRYBB3*, *CRYGB*, *CRYGC*, *CRYGD*, *GRYGS*, *CTDP1*, *CYP27A1*, *EPG5*, *EPHA2*, *EYA1*, *FAM126A*, *FBN1*, *FOXE3*, *FTL*, *FYCO1*, *FZD4*, *GALK1*, *GALT*, *GCNT2*, *GJA1*, *GJA3*, *GJA8*, *HSF4*, *INTS1*, *LEMD2*, *LIM2*, *LSS*, *MAF*, *MIP*, *MYH9*, *NF2*, *NHS*, *OCRL*, *OPA3*, *P3H2*, *PANK4*, *PAX6*, *PITX3*, *RRAGA*, *SIl1*, *SIPA1L3*, *SLC16A12*, *SLC33A1*, *TDRD7*, *UNC45B*, *VIM*, *VSX2*, *WFS1*.

**Figure 1:**
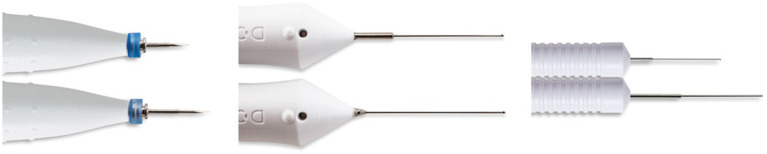
Surgical instruments, D.O.R.C. 27G Ultrashort Vitrectomy kit (above) compared to D.O.R.C. standard 27G instruments (below). From left to right, one-step canula system, TDC vitrectome and endoillumination probe. With permission from D.O.R.C. International BV, Zuidland, the Netherlands.

All cataract surgeries were performed with patients under general anaesthesia by a single surgeon (FN) using the D.O.R.C. EVA Phaco Vitrectomy platform (Dutch Ophthalmic Research Centre International BV, Zuidland, the Netherlands) using the ultra-short 27G vitrectomy kit comprising the 27G Ultra Short One-Step cannula system and 27G Ultra Short Two-Dimensional Cutting (TDC) Cutter. Surgical visualisation was achieved using the ZEISS OPMI LUMERA 700 ophthalmic surgical microscope (Carl Zeiss AG, Oberkochen, Germany) which has been supplemented by the NGENUITY 3D visualisation system (Alcon Inc., Geneva, Switzerland) since 2019. Two to three trocars were placed via clear cornea incisions, one superiorly, one temporally for the infusion and, when required, an additional trocar superonasally/nasally. After filling the anterior chamber with cohesive viscoelastic (HEALON PRO 1%, Johnson & Johnson Vision, Irvine, CA, United States), a small anterior capsulotomy large enough to facilitate access to the lens was made using the cutter. Cataract extraction was performed with the device in flow mode under core vitrectomy settings: 12.000 cuts/min, aspiration at 10–15 cc/min with maximal suction limited to 650 mmHg, irrigation at 20 mmHg initiation bottle pressure with linear compensation to maximally 30 mmHg. Lens cortical remnants, if any, were removed at low aspiration rate. Upon completion, a small posterior capsulotomy was fashioned with the cutter. Viscoelastic was injected for posterior displacement of the vitreous face. Enlargement of both the anterior and posterior capsulotomy is then performed to approximately 6 mm and 4–5 mm in diameter, respectively. For children over the age of 2, concurrent implantation of a monofocal intraocular lens (IOL) was performed. IOL power was calculated using the SRKII formula and post IOL implant refractive targets were selected based on the age at the time of implantation using the rule of seven as described by Enyadi et al. The viscoelastic material is removed and the eye is checked for vitreous prolapse before incision closure. All surgical incisions were sutured using 10–0 Nylon. Sutures were removed under anaesthesia 4 weeks after surgery at which time an objective refraction was performed. The surgical instrumentation is depicted in Figure 1 and a quantitative comparison to D.O.R.C.’s standard 27G instruments can be found in Supplementary Table S1. The surgical procedure is illustrated in an edited video accessible through the Supplementary Video section. Standard post-operative eyedrop regime included tobramycin/dexamethasone (combined formulation) 6 times/day for a week, followed by 4 times/day for 3 weeks, prednisolone acetate 4 times/day tapered over 4–6 weeks and tropicamide twice daily for 2 weeks only.

Post-operative amblyopia management in unilateral cataract cases commences as soon as possible, usually shortly after suture removal. This requires a clear visual axis and adequate optical correction. Occlusion of the unoperated eye usually ranges from 1 h a day up to half of the waking hours a day up to at least 5 years of age. Adaptations to the regimen are guided by the visual acuity development observed. Compliance is often challenging. Close monitoring complemented with parental education, guidance and support throughout the postoperative visual rehabilitation period are critical to maximise the visual potential. Occlusion therapy is also necessary in bilateral cataract cases with an acuity difference. The regimen in these cases is determined on a case-by-case basis.

This study had the relevant research ethics committee approval (Commission of Medical Genetics of Ghent University Hospital and Ghent University) with the assigned code BC-10687 and adhered to the tenets of the Declaration of Helsinki.

## 3. Results

### 3.1. Demographics and presenting clinical features

This retrospective study identified 52 paediatric patients (72 eyes), of which 22 (42.3%) were female and 30 (57.7%) were male. Patient age ranged from 5 weeks to 13 years at the time of (first) surgery (mean 2.9 years): 12 (23.1%) were <6 months of age, 6 (11.5%) were 6 to <12 months of age, 7 (13.5%) were 1 to <2 years of age and 27 (51.9%) were ≥2 years of age.

At presentation, 12 (23.1%) had nystagmus and 35 (67.3%) had strabismus. Preoperative VA recordings were deemed inaccurate or could not be reliably quantified in 24 eyes (33.3%) of 19 children aged < 2 years. In this age group, VA assessment could be achieved in 8 eyes (11.1%) of 5 children and ranged from 0.81 to 2.60 LogMAR. Preoperative VA in children aged 2 to < 6 years could not be retrieved in 5 eyes (6.9%) of 3 children and VA ranged from 0.38 to 2.28 LogMAR in the remaining 19 eyes (26.4%) of 12 children. In the 16 eyes (22.2%) of 12 children aged >6 years, preoperative VA ranged from 0.24 to 3.00 LogMAR.

For the purpose of visual outcome analysis, pre-and post-operative visual acuity data were categorised into group 1: bilateral cataract, group 2: unilateral cataract, group 3: secondary cataract. In group 1 (*n* = 39, 54.2%), preoperative VA ranged from 0.24 to 1.98 LogMAR and could not be determined or was unquantifiable in 12 eyes. In group 2 (*n* = 18, 25.0%), VA ranged from 0.40 to 2.60 LogMAR and was unquantifiable in 7 eyes. In group 3 (*n* = 15, 20.8%), VA ranged from 1.70 to 3.00 LogMAR and was unquantifiable in 10 eyes. Group 3 included cataracts secondary to or associated with posterior segment disease, including PFV, or secondary to vitrectomy. In this group, lens extraction was often performed to facilitate visualisation and management of the underlying pathology. For the case with Marfan syndrome (0200), preoperative VA could not be determined. See Table 1 for a case-by-case overview of these parameters.

**Table 1:** Demographic details and clinical characteristic of 52 patients who underwent paediatric cataract surgery at the Ghent University Hospital.

Patient ID	Laterality	OD/OS	Pre-op VA (OD, OS) (LogMAR)	Strabismus	Nystagmus	Cataract subtype	Aetiology	Additional findings
0101	U	OD	1.70	+	−	Nuclear	Developmental	Microphthalmia
								A-P PFV

0200	B	OU	CND	−	−	(Lens subluxation)	Lens subluxation	High axial length
							Marfan syndrome (*FBN1*)	Marfanoid built
								Hypermobility
								Aortic root dilation
0301	U	OD	HM (2.28)	−	−	Nuclear	Secondary	High axial length Bilateral giant retinal tears requiring multiple treatments including bilateral vitrectomy
							Stickler syndrome (*COL2A1*)	
0400	B	OU	1,70	−	−	Anterior polar	Genetic	IUGR
								Microphthalmia and unilateral ptosis (OS)
							IDDDFP (*BRPF1*)	Facial dysmorphia
								Mild intellectual delay
0500	B	OU	0.65, 0.65	+	−	Nuclear	Familial	Isolated bilateral cataract with positive family history
								No genetic testing done
0602	B^Δ^	OS	HM (2.28)	+	−	Nuclear	Unknown	Visually insignificant cataract in the other eye
								Negative family history
								No genetic testing done
0800	B	OU	1.52	−	+	Nuclear	Familial	Bladder exstrophy
								Bilateral cataract with positive family history
								No genetic testing done
0901	U	OD	0.40	−	−	Posterior subcapsular	Idiopathic	
1000	B	OU	0.66, 0.56	+	−	Posterior subcapsular	Secondary	Steroid-resistant nephrotic syndrome
							High-dose systemic steroids	
1102	U	OS	1.30	−	−	Cortical and posterior subcapsular	Idiopathic	
1300	B	OU	DNFF	+	+	Nuclear	Unknown	Isolated bilateral cataract
								*De novo* heterozygous VUS in *FOXE3*
								*De novo* 1.3 Mb CNV within 20q21.21
1401	U	OD	1.48	+	−	Posterior subcapsular	Idiopathic	
1500	B	OU	0.56, 0.58	−	−	Lamellar	Unknown	Consanguineous marriage
								Negative family history
								WES negative for cataract genes
1600	B	OU	0.90	+	−	Nuclear	Genetic	Heterozygous VUS *SIPA1L3*
							AD Cataract (*CRYAA*)	
1701	U	OD	FF	+	+	Nuclear	Idiopathic	
1800	B	OU	CND	+	−	Nuclear	Developmental	Facial dysmorphia
							Hallermann-Streiff Syndrome	Dental anomalies
								Mandibular
								hypoplasia
								Small stature
								Hypoplastic skin
								Hypotrichosis
1900	B	OU	FF	+	−	Posterior subcapsular	Genetic	
							Down syndrome	
2002	B ^Δ^	OS	DNFF	+	+	Unspecified	Secondary	Liver failure and encephalopathy in neonatal period
							Galactosaemia (*GALT*)	Successful dietary management of cataract Bilateral vitreous haemorrhage requiring vitrectomy
2100	B	OU	CND	+	−	Cortical	Genetic	Anterior synechiae and corneal leucoma (OD)
							Anterior segment dysgenesis (*PITX3*)	Bilateral microcornea Negative family history
2200	B	OU	0.68, 0.68	+	−	Posterior subcapsular	Unknown	No genetic testing done
2302	U	OS	DNFF	+	+	Nuclear and posterior subcapsular	Idiopathic	
2402	U	OS	CND	−	+	Nuclear and posterior subcapsular	Idiopathic	
2500	B	OU	0.36, 0.38	−	−	Cortical (OD), sutural and cortical (OS)	Genetic	Isolated bilateral cataract
							X-linked cataract (*NHS*)	
2601	U	OD	1,13	+	−	Nuclear	Unknown	Isolated unilateral cataract Heterozygous VUS in *NHS* Bilateral dense congenital cataract in brother and mother but negative history in grandfather and all are heterozygous for the same variant
2701	U	OD	0.70	−	−	Posterior polar	Idiopathic	
2801	U	OD	1.3	−	−	Nuclear	Idiopathic	
2900	B	OU	0.52, 0.48	−	−	Nuclear and posterior polar	Familial	Positive family history
								WES negative for cataract genes
3000	B	OU	0.38, 0.38	+	−	Nuclear	Genetic	FTT
							1q21.1 microdeletion syndrome	Global developmental delay Telecanthus
3101	U	OD	FF	+	−	Nuclear and posterior polar	Idiopathic	
3200	B	OU	*CF* (1.98), 0.74	+	−	Anterior subcapsular (OD), anterior and posterior subcapsular (OS)	Unknown	Negative family history
								WES negative for cataract genes
3301	U	OD	0.81	+	−	Anterior subcapsular	Secondary	Xanthogranulomatosis of scalp and iris (OD) with spontaneous hyphaema and anterior uveitis
							Juvenile xanthogranulomatosis	
3402	U	OS	LP (2.60)	+	−	Unspecified	Idiopathic	Heterozygous VUS *CRYBA4*
3501	U	OD	CND	+	+	Unspecified	Idiopathic	
3601	U	OD	CND	+	−	Unspecified	Developmental	Chorioretinal coloboma
								Optic nerve head hypoplasia of same eye
								Negative family history
								WES negative for ASD genes
3701	B^Δ^	OD	LP (2.60)	+	+	Unspecified	Secondary	Insignificant cataract in the other eye
							Leber congenital amaurosis (*GUCY2D*)	
3801	B^Δ^	OD	LP− (3.00)	+	+	Unspecified	Secondary	Insignificant cataract in the other eye
							Leber congenital amaurosis (*NMNAT1*)	
3900	B	OU	CND	+	+	Unspecified	Genetic	
							AD Cataract (*MIP*)	
4101	U	OD	CND	+	−	Unspecified	Developmental	Microphthalmia of same eye
								Negative family history
								WES negative for cataract genes
4201	U	OD	HM (2.28)	+	−	Unspecified	Developmental	Microcornea of same eye
								Negative family history
								No genetic testing done
4302	U	OS	CND	+	+	Unspecified	Idiopathic	Giant omphalocoele
								Septal defects of heart not requiring treatment
								Heterozygous VUS *EYA1*
4400	B	OU	1.13, 1.26	+	−	Nuclear	Unknown	IUGR
								Hypotonia
								Developmental delay, Severe autism
								Negative family history
								WES negative for ID genes Cataract genes not tested
4501	U	OD	0.54	−	−	Posterior polar	Idiopathic	
4600	B	OU	0.32, 0.24	−	−	Sutural	Idiopathic	Isolated bilateral cataract Negative family history
								WES negative for cataract genes
4701	B^ΔΔ^	OD	CND	−	−	Unspecified	Secondary	Dental anomalies
							Incontinentia pigmenti (*IKBKG*)	Stroke
								Retinal detachment (OD) requiring vitrectomy
4802	B^Δ^	OS	CND	+	+	Nuclear and posterior subcapsular	Secondary	Prematurity (GA 24 weeks)
							Severe prematurity	BPD, NEC, Hydronephrosis, Periventricular leukomalacia Stagnant postnatal ocular development and growth Asymmetric cataract
								ROP and funnel shaped retinal detachment (OS)
								Heterozygous VUS *WFS1*
4902	U	OS	CND	+	−	Posterior subcapsular	Secondary	Retinal detachment requiring vitrectomy
							Retinoschisis (*RS1*)	
5001	U	OD	CND	−	−	Unspecified	Developmental	A-P PFV
5101	U	OD	0.42	+	−	Unspecified	Secondary	Asymmetric buphthalmos requiring trabeculectomy (OU) and multiple bleb revisions (OD)
							Congenital glaucoma	WES negative for ASD genes
5201	U	OD	DNFF	+	−	Nuclear and posterior polar	Developmental	A-P PFV
5300	B	OU	CND	−	−	Unspecified	Secondary	Bilateral total retinal detachment requiring vitrectomy
							Norrie disease (*NDP*)	
5402	U	OS	LP− (3.00)	+	−	Posterior subcapsular	Secondary	Bilateral multifocal retinoblastoma requiring chemotherapy, transpupillary thermotherapy, cryotherapy
							Retinoblastoma (*RB1*)	Complicated by chronic total retinal detachment (OS)
5502	U	OS	CND	+	−	Nuclear	Secondary	Total retinal detachment requiring vitrectomy
							Morning glory syndrome	

U, Unilateral cataract; B, Bilateral cataract; B^Δ^, Only unilateral surgery in bilateral cataract cases; B^ΔΔ^, Bilateral surgery, but only one eye met inclusion criteria; OD, lens extraction oculus dexter; OS, lens extraction oculus sinister; OU, lens extraction both eyes; CND, could not be determined; FF, fixates and follows; DNFF, does not fixate and follow; CF, counting fingers; HM, hand motion; LP−, no light perception; +, Present; −, Absent; A-P PFV, Combined Anterior and Posterior Persistent Foetal Vasculature; IDDDFP, Intellectual developmental disorder with dysmorphic facies and ptosis; IUGR, intrauterine growth retardation; FTT, Failure to thrive; GA, gestational age; NEC, necrotising enterocolitis; BPD, bronchopulmonary dysplasia; ASD, anterior segment dysgenesis; ID, intellectual disability; WES, whole exome sequencing; VUS, variant of unknown significance.

Axial length (AL) data could not be retrieved for 19 eyes (26.4%) of 14 patients. In patients aged < 6 months (*n* = 7), AL ranged from 15.71 to 23.98 mm (median 19.08 mm). In patients aged 6 to < 1 year (*n* = 4), AL ranged from 16.65 mm to 18.72 mm (median 17.88 mm). In patients aged 1 to < 2 years (*n* = 6), AL ranged from 17.04 to 23.14 mm (median 19.69 mm). In patients aged > 2 years old (*n* = 21), AL ranged from 18.88 to 25.00 mm (median 21.99 mm).

Pre-operative IOP ranged from 5.0–22.4 mmHg (median 12.8 mmHg).

**Figure 2:**
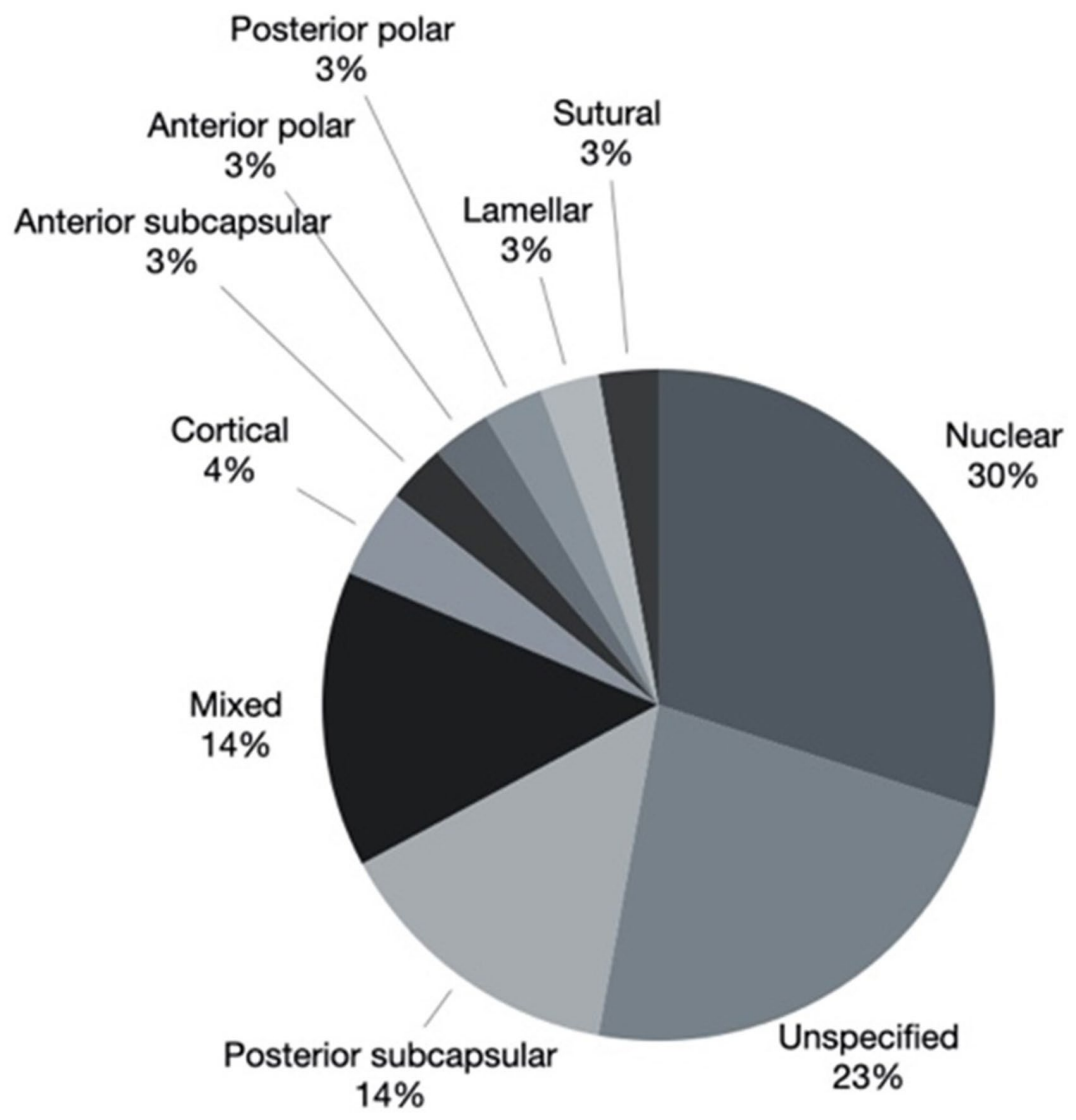
Cataract morphology of 63 eyes. *2 eyes where clear lens extraction was performed for lens subluxation associated with Marfan syndrome were excluded.

Cataract morphology was documented as: nuclear in 19 eyes (27.1%) of 13 patients, posterior subcapsular in 11 eyes (15.7%) of 8 patients, mixed in 9 eyes (12.9%) of 8 patients, cortical in 3 eyes (4.3%) of 2 patients, anterior subcapsular in 2 (2.9%) eyes of 2 patients, anterior polar in 2 (2.9%) eyes of 1 patient, posterior polar in 2 eyes (2.9%) of 2 patients, lamellar in 2 eyes (2.9%) of 1 patient, sutural in 2 (2.9%) eyes of 1 patient, unspecified in 18 eyes (25.7%) of 15 patients. The mixed group was composed of nuclear and posterior polar components in 4 eyes of 3 patients and nuclear and posterior subcapsular components in 2 eyes of 2 patients. The combinations sutural with cortical, cortical with posterior subcapsular and anterior subcapsular with posterior subcapsular were recorded in 1 eye each. Cataract morphology is represented graphically in Figure 2.

### 3.2. Cataract aetiology

**Figure 3:**
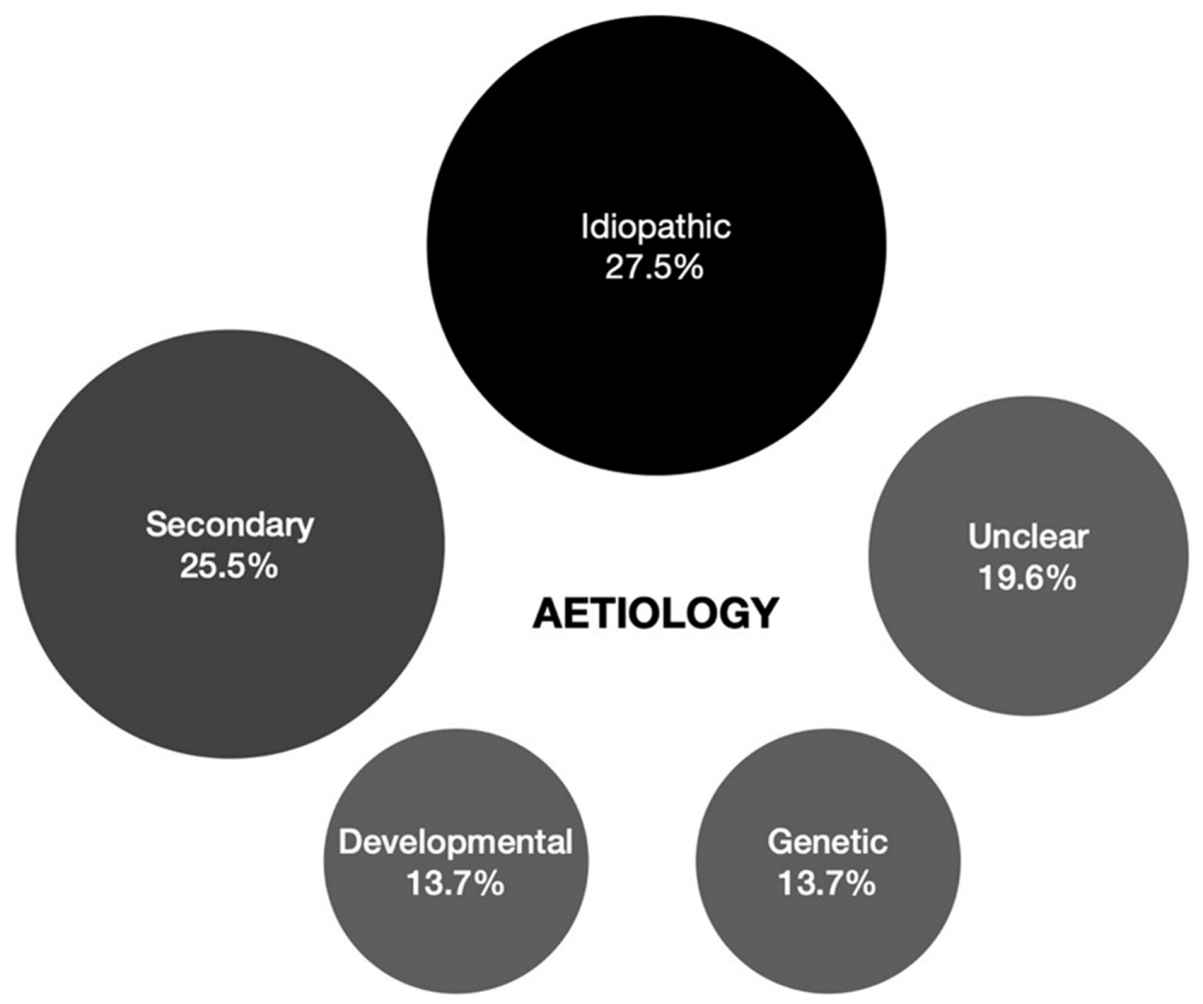
Overview of cataract aetiology of 63 eyes which underwent paediatric cataract surgery at the Ghent University Hospital. *2 eyes where clear lens extraction was performed for lens subluxation associated with Marfan syndrome were excluded.

Causes underlying cataract in this cohort were identified to be secondary in 13 (25.5%), developmental in 7 (13.7%), and genetic in 7 cases (13.7%). In 14 (27.5%) isolated, mostly unilateral cataract cases no cause could be identified despite extensive work-up and were deemed idiopathic. In 3 bilateral cataract cases (5.9%) no definite cause had been identified at the time of the study but were deemed familial based on family history. In 7 cases (13.7%) it was improper to designate them to one of the aetiologic categories due to contradicting or insufficient information. When no genetic analysis was performed, this was due to parents’ preference. The different aetiologies are represented graphically in Figure 3.

#### 3.2.1. Secondary cataract

In this cohort, secondary cataract was defined as cataract not directly considered part of the primary underlying condition. These include cataracts that developed following medical intervention for the primary condition or that are known complications of the primary condition. The secondary cataract subgroup comprised steroid-induced (*n* = 1), juvenile xanthogranulomatosis (JXG; *n* = 1), congenital glaucoma (*n* = 1), galactosaemia (*n* = 1; genetically confirmed), incontinentia pigmenti (*n* = 1; genetically confirmed), retinopathy of prematurity (ROP; *n* = 1; gestational age 24 weeks), morning glory syndrome (*n* = 1), Norrie disease (*n* = 1; genetically confirmed), retinoblastoma (*n* = 1; genetic predisposition confirmed), retinoschisis (*n* = 1; genetically confirmed), Stickler syndrome (*n* = 1; genetically confirmed), and Leber congenital amaurosis (LCA; *n* = 2; genetically confirmed).

The case of JXG (3301) developed unilateral cataract in the eye with a large iris xanthogranuloma causing spontaneous, non-resolving hyphaema and anterior chamber inflammation necessitating anterior chamber washout. The 7-year-old with congenital glaucoma (5101) underwent bilateral trabeculectomies and cataract developed in the eye requiring multiple bleb revisions. No genetic explanation for the glaucoma was found using whole exome sequencing. The 2-month-old with galactosaemia (2002) had bilateral mild lens opacification and total vitreous haemorrhage (VH) at presentation. Both cataracts resolved with dietary adaptation, but vitrectomy was required for persistent VH. Cataract extraction with IOL implant was performed for unilateral (left) visually significant cataract 2 months after vitrectomy. This case was further complicated by progressive liver failure and encephalopathy during the neonatal period. The cases of incontinentia pigmenti (4701), ROP (stage 4; 4802), morning glory syndrome (5502), Norrie disease (5300), retinoblastoma (5402), retinoschisis (4902) and Stickler syndrome (0301) all underwent vitrectomy for retinal detachment with sequential cataract surgery at a separate timepoint for secondary cataract. Cataract surgery was performed in the case with retinoblastoma to facilitate tumour surveillance. Two unrelated cases (3701 and 3801) of LCA due to mutations in *GUCY2D* (MIM *600179) and *NMNAT1* (MIM *608700), were both diagnosed with unilateral (right eye) visually significant cataract of at the age of 6, whilst the contralateral eye showed mild cataract of no visual significance. LCA is not typically associated with cataract. A 3-year-old (0200) with genetically confirmed Marfan syndrome underwent bilateral clear lens extraction for visually significant lens subluxation and was excluded from Figures 2, 3. The molecular diagnoses at the basis of the monogenic secondary cataract cases as well the Marfan syndrome case are displayed in Table 2.

**Table 2:** Genotype and phenotype correlation for surgical cataract cases of genetic and metabolic aetiology as well as cases treated for secondary cataract with genetic conditions which may or may not be typically associated with early-onset cataract.

ID	Gene (RefSeq)	Variant	Variant type	Zygosity	Methodology	Segregation/family history	MOI	Phenotype (MIM)	Patient phenotype match	Reference (PMID)	ClinVar ID	Allele frequency gnomAD (v3.1.2)	*In-silico* analysis	ACMG classification
Primary isolated or syndromic cataract														
0400	*BRPF1* (NM_001003694.2)	c.2546del, p.(Gly849Valfs*3)	Frameshift	Het	Not known (executed at different centre)	*De novo*/negative	AD	Intellectual developmental disorder with dysmorphic facies and ptosis (617333)	Typical	Novel	NA	NA	NA	Pathogenic
1600	*CRYAA* (NM_000394.3)	c.61C > T, p.(Arg21Trp)	Missense	Hom	WES	Not possible due to pseudogene/negative	AR	Cataract 9, multiple types (604219)	Typical	Hansen L et al. Invest Ophthalmol Vis Sci 2007 (17724170)	68460	0.00002413	REVEL: 0.890	Likely pathogenic
1900	N/A	Trisomy 21	Trisomy	NA	Karyotyping	NA	NA	Down syndrome (190685)	Typical					
2100	*PITX3* (NM_005029.3)	c.640_656dup, p.(Gly220Profs*95)	Frameshift	Het	WES	Not done/negative	AD	Anterior segment dysgenesis 1, multiple subtypes (107250)	Typical	Semina EV et al. Nat Genet 1998 (9620774)	468353	NA	NA	Likely Pathogenic
2500	*NHS* (NM_198270.4)	c.3803_3806dup, p.(Gln1270Asnfs*29)	Frameshift	Hemi	WES	*De novo*/negative	XLD	Cataract 40, X-linked (302200)	Typical	Novel	NA	NA	NA	Likely Pathogenic
3000	N/A	1q21-1q21.2 1.3 Mb deletion	Large deletion	Het	Array CGH	Present in affected half-sister and mother	AD	Chromosome 1q21.1 deletion syndrome (612474)	Typical					
3900	*MIP* (NM_012064.4)	c.97C > T, p.(Arg33Cys)	Missense	Het	WES	Present in affected mother and grandmother	AD	Cataract 15, multiple types (615274)	Typical	Gu F et al. Case Reports 2007 (17893667)	217342	NA	REVEL: 0.763	Likely Pathogenic
Variants of unknown significance														
1300	*FOXE3* (NM_012186.3)	c.571 T > G, p.(Tyr191Asp)	Missense	Het	WES	*De novo*/negative	AD AR	Anterior segment dysgenesis 2, multiple subtypes (610256)	Possibly	Novel	NA	NA	REVEL: 0.242	VUS
1701	*SIPA1L3* (NM_015073.3)	c.4186C > T, p.(Arg1396*)	Nonsense	Het	WES	Not done/negative	AD or AR (missing second?)	?Cataract 45 (616851)	Atypical: unilateral cataract	Novel	NA	NA	NA	VUS
2601	*NHS* (NM_198270.4)	c.478A > G, p.(Thr160Ala)	Missense	Het	WES	Affected brother and mother heterozygous	XLD	Nance-Horan syndrome (302350)	Atypical: unilateral cataract	Novel	NA	NA	REVEL: 0.058	VUS
3402	*CRYBA4* (NM_001886.2)	c.511G > A, p.(Gly171Ser)	Missense	Het	WES	Not done/negative	AD?	Cataract 23 (610425)	Atypical: unilateral cataract	Wang Z et al. Plos one 2020 (31935276)	NA	0.00003284	REVEL: 0.762	VUS
4302	*EYA1* (NM_000503.6)	c.590C > T, p.(Thr197Ile)	Missense	Het	WES	Father heterozygous, absent in mother/negative	AD?	Anterior Segment anomalies with or without cataract (602588)	Atypical: unilateral cataract	Novel	NA	NA	REVEL: 0.420	VUS
4802	*WFS1* (NM_006005.3)	c.683G > A, p.(Arg228His)	Missense	Het	WES (RetNet)	Not done/negative	AD	Cataract 41 (116400)	Atypical: unilateral cataract	Valéro R et al. Diabet Med 2008 (18544103)	198190	0.0008538	REVEL: 0.625	VUS
Secondary cataract and other														
0200	*FBN1* (NM_000138.5)	c.5546-2A > C	Splice acceptor site	Het	Not known (executed at different centre)	Not known	AD	Marfan syndrome (154700)	Typical	Novel	NA	NA	Loss of acceptor site	Pathogenic
0301	*COL2A1* (NM_001844.5)	c.1957C > T, p.(Arg653*)	Nonsense	Het	NGS of *COL2A1*, *COL11A1* and *COL11A2*	*De novo*/negative	AD	Stickler syndrome, type I, nonsyndromic ocular (609508)	Typical	Liberfarb RM et al. Genet Med 2003 (12544472)	17395	NA	NA	Pathogenic
2002	*GALT* (NM_000155.3)	c.563A > G, p.(Gln188Arg)	Missense	Hom	WES	Not done/negative	AR	Galactosaemia (230400)	Typical	Reichardt JK et al. Am J Hum Genet 1991 (1897530)	3614	0.001866	REVEL: 0.975	Pathogenic
3701	*GUCY2D* (NM_000180.3)	c.2773G > T, p.(Glu925*)	Nonsense	Hom	Homozygosity mapping + Sanger sequencing of *GUCY2D*	Parents carrier/confirmed in affected sibling	AR	Leber congenital amaurosis 1 (204000)	Typical	This patient’s novel genotype was reported in Hahn L C et al. Ophthalmol Retina 2022 (35314386)	NA	NA	NA	Pathogenic
3801	*NMNAT1* (NM_022787.4)	c.679C > T, p.(Arg227Trp)	Missense	Het	Sanger sequencing of *NMNAT1*	Parents carrier/negative	AR	Leber congenital amaurosis 9 (608553)	Typical	Coppieters F et al. Human Mutat 2015 (26316326)	NA	0.000006574	REVEL: 0.942	Likely Pathogenic
		c.769G > A, p.(Glu257Lys)	Missense	Het						22842227, 22842229, 22842,30, 22842231	37134	0.0008149	REVEL: 0.694	Pathogenic
4701	*IKBKG* (NM_003639.3)	Exon 4–10 deletion	Deletion	Het	Long range PCR (at different centre)	Not done/negative	XLD	Incontinentia pigmenti (308300)	Typical	Smahi A et al. Nature 2000 (10839543)	11447	NA	NA	Pathogenic
4902	*RS1* (NM_000330.4)	c.433G > C, p.(Asp145His)	Missense	Hemi	NGS of *RS1*	Not done/negative	XLR	Retinoschisis (312700)	Typical	Li X et al. Mol Vis 2007 (17615541)	NA	NA	REVEL: 0.916	Likely pathogenic
5300	*NDP* (NM_000266.4)	c.220C > T, p.(Arg74Cys)	Missense	Hem	NGS and MLPA: *FZD4*, *LRP5*, *NDP* NGS: *TSPAN12*	Mother heterozygous/negative	XLR	Norrie disease (310600)	Typical	Berger W et al. Hum Mol Genet 1992 (1307245)	167326	NA	REVEL: 0.87	Pathogenic
5402	*RB1* (NM_000321.3)	c.1285A > T, p.(Lys429*)	Nonsense	Het	NGS and MLPA of *RB1*	Not done/negative	AD	Retinoblastoma (180200)	Typical	Novel	NA	NA	NA	Pathogenic

Variants of unclear significance are indicated separately. Het, Heterozygous; Hom, Homozygous; Hem, Hemizygous; VUS, Variant of uncertain significance; WES, Whole exome sequencing; AD, Autosomal dominant; AR, Autosomal recessive; XLD, X-linked dominant; XLR, X-linked recessive; RefSeq, Reference sequence; NGS, Next Generation Sequencing; MLPA, Multiplex Ligation-dependent Probe Amplification; PCR, Polymerase Chain Reaction; MOI, mode of inheritance.

#### 3.2.2. Genetic and metabolic

Genetic variants that were considered likely explanatory for the phenotype were found in 5 cases (9.8%) using the cataract panel: 1 in *NHS* (MIM *300457), 1 in *BRPF1* (MIM *602410), 1 in *CRYAA* (MIM *123580), 1 in *MIP* (MIM *154050), 1 *PITX3* (MIM *602669).

Case 2002 had primary bilateral cataract and multisystemic disease determined to be due to a homozygotic missense mutation in *GALT* (MIM *606999) which was identified after cataracts were noticed during an ophthalmological assessment for suspicion cystinosis. This case is discussed amongst the secondary cataract cases. Molecular cytogenetics using comparative genomic hybridisation identified 1q21.1 microdeletion syndrome in one patient (3200) with bilateral congenital cataracts, failure to thrive and mild neuromotor delay. The affected mother and sister were shown to have a genomic anomaly. Karyotyping identified trisomy 21 in a patient (1900) with clinical features of Down syndrome. Genotype–phenotype correlation data are displayed in Table 2.

#### 3.2.3. Developmental

In this cohort, developmental cataract was defined as cataract complicated by ocular and/or extraocular developmental anomalies and for which there was no monogenic explanation. In 3 cases (42.9%) the cataract was part of PFV syndrome. A single case (14.3%) had a diagnosis of Hallermann-Streiff-François Syndrome who showed facial dysmorphia, dental anomalies, mandibular hypoplasia, cutaneous hypoplasia, hypotrichosis, and small stature in addition to bilateral congenital cataract. The remaining 3 cases (42.9%) were unilateral cataracts associated with additional ocular anomalies such as microphthalmia, but excluding PFV.

### 3.3. Surgical outcomes and amblyopia management

The average surgical duration, taken from knife-to-skin to skin closure, was 51.8 min (range 24–70 min) per eye. Accuracy of the recorded timing could not be verified with retrospective review of the surgical log. A general trend towards shorter duration over time was observed.

Bilateral cataract extraction was performed in 20 (38.5%) patients, of which 13 (25%) were concurrent same-day surgery, 7 (13.5%) were sequential within 14 days to 14 weeks apart depending on the severity of the contralateral cataract. Twenty-three (44.2%) patients (aged 2.0 to 12.7 years, median 5.5 years) had primary IOL implantation. In these patients, a single-piece acrylic IOL, the Acrysof SN60WF (Alcon Inc., Geneva, Switzerland), was implanted through a 2.2 mm clear corneal incision. Eleven (21.2%) patients (aged 1.1 to 7.4 years, median 2.8 years) underwent secondary IOL implantation of which 2 were younger than 2 years at the time of implantation. A 3-piece IOL, the MA50BM (Alcon Inc., Geneva, Switzerland), was implanted in the sulcus for all cases. Patient 4,302 underwent unilateral cataract surgery at 2 months of age and secondary IOL implantation was performed just after his first birthday (1.1 years). Patient 1900 underwent bilateral cataract extraction at 9 months of age and secondary IOL implantation about 4 months short of his second birthday (1.7 years). Eighteen patients (34.6%), aged 32 days to 10.1 years (median 36 weeks), were still aphakic at the time of analysis. Fourteen (26%) were < 2 years old and 4 (7.7%) were ≥ 2 years old. In the latter cohort, cases that were left aphakic included lens subluxation due to Marfan syndrome (0200), microcornea (2200), retinal detachment and haemorrhage (4902), and very low vision (no light/dark perception as measured with Berkeley Rudimentary Vision Testing) due to retinoblastoma (5402).

#### 3.3.1. Visual outcomes

In group 1 (*n* = 39, 54.2%), postoperative VA ranged from −0.04 to 2.24 LogMAR. In group 2 (*n* = 18, 25.0%), VA ranged from 0.0 to 2.28 LogMAR and was unquantifiable in 4 eyes. In group 3 (*n* = 15, 20.8%), VA ranged from 0.40 to 3.00 LogMAR and was unquantifiable in 6 eyes. For the case with Marfan syndrome (0200), postoperative VA were 0.32 and 0.6 LogMAR in his right and left eye, respectively.

**Figure 4:**
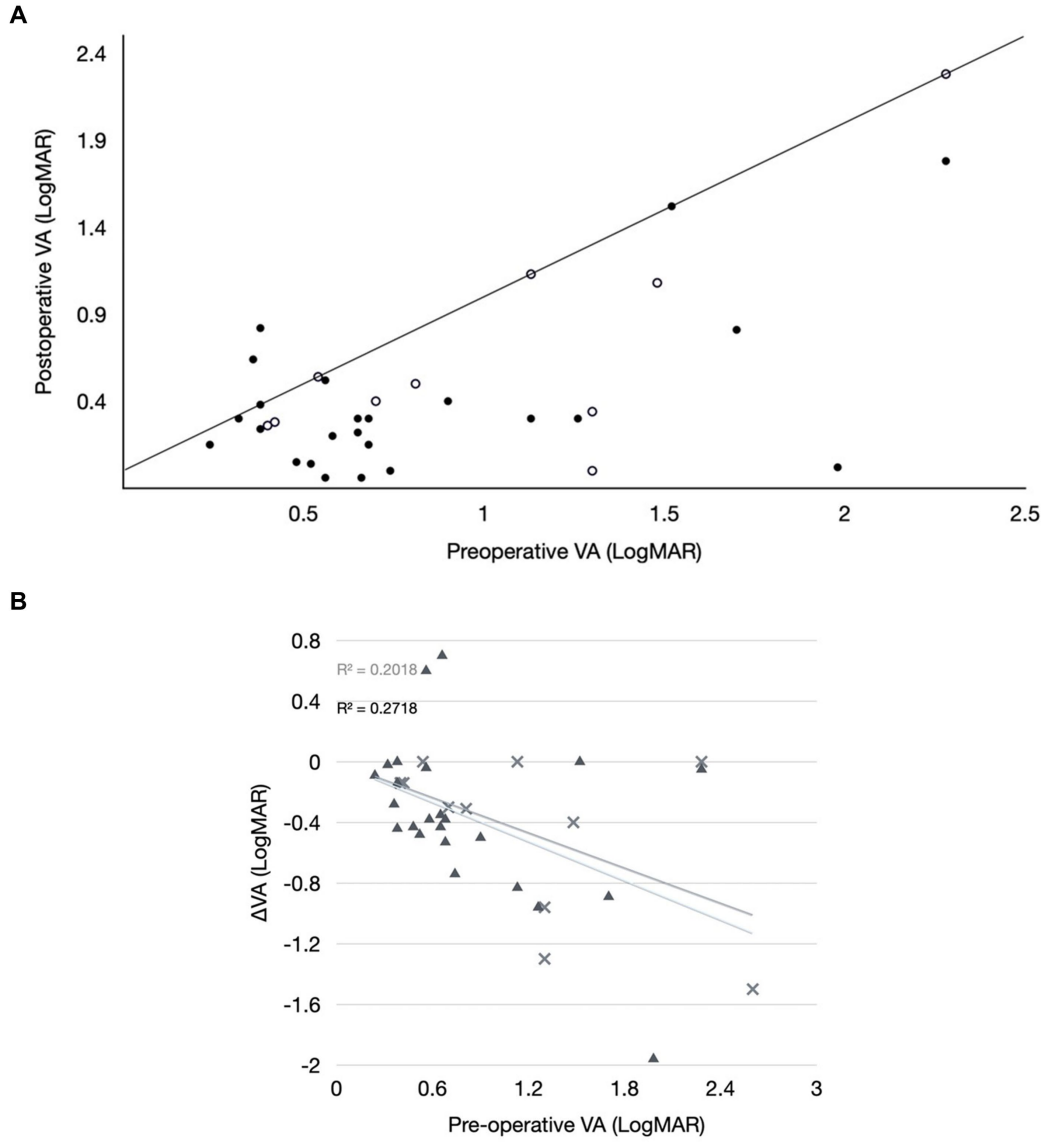
**(A)** Comparison of pre-operative and post-operative visual acuities (VA). Included are 27 eyes (full black dots) of patients with bilateral cataracts, 11 eyes (circles) of patients with unilateral cataracts. The continuous line (line of no change) separates eyes with worse (left upper triangle) and better (right lower triangle) VA. All cases with ocular comorbidities confounding VA and all cases with incomparable or unquantifiable VA were excluded from this graph. **(B)** Visual acuity (VA) gains or losses (ΔVA) for the VA pairs of **(A)** with pre-operative VA plotted as the independent variable.

Postoperative visual acuities were recorded at various time points. In the chart comparing pre-and postoperative acuities (Figure 4A), the latter was obtained at the time of postoperative visual stabilisation but prior to amblyopia therapy. Patients in whom VA assessments could not be performed or were unquantifiable, e.g., fixing and following, were excluded. Eyes with PFV and/or retinal disease were also excluded, leaving 38 eyes (52.8% of total). Figure 4B compares VA gains in bilateral vs. unilateral cataract cases and showed no clear difference between both groups. As preoperative VA gets worse, a larger gain from surgery alone can be expected. One patient (2500) had an initial drop in VA in both eyes following surgery without any complication to explain for this, but the most recent VA recording showed an overall net VA gain in both eyes compared to the pre-operative VA. Overall, 23 of these 38 eyes (60.5%) had a minimal VA gain of −0.2 LogMAR (= two rows or 10 letters on EDTRS).

#### 3.3.2. Complications

**Figure 5:**
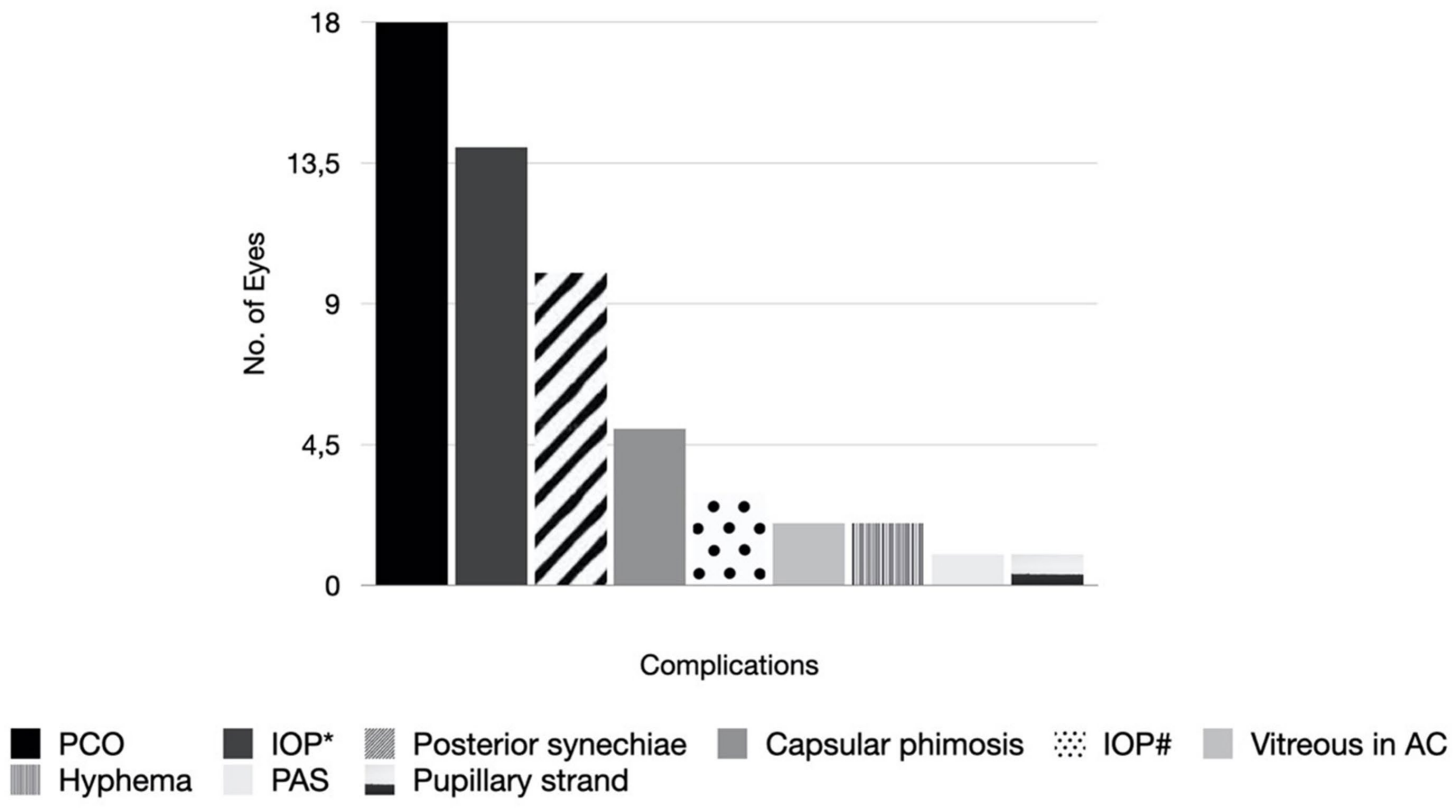
Complications after cataract surgery. PCO = Posterior capsule opacification. IOP* = IOP rise requiring pharmacological treatment. IOP# = IOP rise requiring surgical intervention. Vitreous in AC = Vitreous in anterior chamber. PAS = Peripheral anterior synechiae.

No significant perioperative complications were recorded. Post-operative complications were reported in 44 (61.1%) eyes, 18 (41.0%) posterior capsule opacification (PCO), 14 (31.8%) IOP rise successfully managed with pharmacological treatment, 3 (6.8%) IOP rise requiring surgical intervention, 10 (22.7%) posterior synechiae formation, 5 (11.4%) capsular phimosis, 2 (4.5%) vitreous in the anterior chamber, 2 (4.5%) hyphaema, 1 (2.3%) peripheral anterior synechiae (PAS) formation and 1 (2.3%) pupillary strand (Figure 5). All cases with PCO required at least one posterior capsulotomy enlargement and 4 eyes (22.2%) of 2 patients required one further repeat procedure. All cases with posterior synechiae formation required synechiolysis. One child (5201) required one further repeat procedure. All cases with capsular phimosis required surgical enlargement. All cases with vitreous in the anterior chamber required surgical removal of the vitreous. No associated retinal traction was reported in those cases. Hyphaema was present on the first post-operative day in the patient with incontinentia pigmenti (4701) which spontaneously resolved by day 4. The aetiology underlying the second case (4902) of hyphaema was unclear, but bleeding resolved spontaneously within 3 days without further complications. The 5-month-old (5300) with PAS required synechiolysis. The 4-year-old (4501) with a pupillary strand across the visual axis required surgical removal of the strand.

The 14 eyes with medically controlled post-operative transient IOP rise belonged to 12 patients (aged 47 days to 12.4 years, median 2.3 years). Five patients had undergone bilateral lens extraction 2 of whom had remained aphakic. Seven had had unilateral lens extraction 4 of whom had remained aphakic. Of these 14 eyes, 2 (14.3%) were managed by tapering of topical steroids alone, 1 (7.1%) by a combination of steroid dosage reduction and topical timolol, 2 (14.3%) by topical latanoprost alone, 6 (42.9%) using 2 topical anti-hypertensives, 2 (14.3%) using three topical anti-hypertensives and 1 (7.1%) using 3 topical anti-hypertensives combined with oral acetazolamide. Anti-hypertensive eye drops were required for up to a maximum of 4 weeks in all cases.

A 2-month-old (5001) who underwent unilateral cataract extraction and remained aphakic had a pre-and post-operative IOP of 18.0 mmHg and 3.3 mmHg respectively, both measurements were taken under general anaesthesia. Intra-operative findings included PFV with contraction of the nasal retina towards the vascular stalk and widespread retinal degeneration. No vitrectomy was required. At post-operative month 1, EUA revealed a dense fibrovascular anterior pupillary membrane, 360° posterior synechiae with an elevated IOP of 35.4 mmHg. Following surgical removal of the persistent tunica vasculosa lentis, the post-operative IOP remained elevated at 30.0 mmHg despite antihypertensive eye drops. The patient was subsequently referred to the paediatric glaucoma service at another centre for surgical intervention and continued follow up.

Bilateral cataract extraction without IOL implant, performed on the same day for the 2-month-old patient (1300) with *FOXE3*-related cataract, was uneventful. Pre-and intra-operative IOP were well-controlled with clear corneas and good visualisation throughout the surgery in both eyes. From post-op day 4, IOP was elevated to 50.0 and 55.0 mmHg, right and left respectively, with aphakic pupil block glaucoma being the likely cause. IOP control remained suboptimal despite maximal medical therapy necessitating surgery (posterior synechiolysis, anterior vitrectomy and peripheral iridectomy). Post-operative day 1, the IOP normalised transiently to 13.0 mmHg and 12.0 mmHg right and left, respectively. Subsequent follow-up visits have recorded fluctuating IOPs, range 19.0–33.0 mmHg in the right eye and 18.0–28.0 mmHg in the left eye whilst being maintained on 3 anti-hypertensive eyedrops. Despite bilateral trabeculectomy with mitomycin C, IOP control remained suboptimal necessitating further bilateral glaucoma drainage surgery with Ahmed tube implant.

#### 3.3.3. Amblyopia management

Of the children who underwent unilateral lens extraction without concomitant IOL implantation (*n* = 22), 7 (31.8%) were prescribed contact lenses, 9 (40.9%) used aphakic glasses, 6 (27.3%) were not prescribed refractive correction due to poor vision from underlying retinal disease. Reasons for selecting aphakic glasses over contact lenses included frequent contact lens loss, parents’ preference, and poor tolerance. Four (40.0%) of the 10 children with unilateral lens extraction and primary IOL implantation were prescribed glasses as an additional optical correction for the operated eye. The remaining 6 (60.0%) children did not require additional corrective glasses. Of the children that underwent bilateral lens extraction without concomitant IOL implantation (*n* = 9), 6 (66.7%) had aphakic glasses and 3 (33.3%) had contact lenses. Of bilateral cases with primary IOL implantation (*n* = 11), 9 (81.8%) required additional corrective glasses.

## 4. Discussion

This retrospective study identified 72 eyes of 52 paediatric cataract patients who had required surgical intervention.

To the best of our knowledge, this is the first study describing the use of ultrashort 27G vitrectomy instrumentation in paediatric cataract surgery. Compared to standard 27G instruments, the inserter and trocar are 25% shorter, whilst the TDC (two-dimensional cutting) cutter is 20% shorter with 60% increase in rigidity. These confer several surgical advantages particular to infant eyes. The average infant eye is approximately two-thirds the size of the adult eye with axial lengths of 16.5 mm and 24.0 mm, respectively. Those with concurrent cataract may have significant microphthalmia. In an infant eye, the iris stroma is often hypoplastic relative to the adult situation and is, therefore, relatively hypervascular, they have shallow anterior chamber (AC), immature trabecular meshwork, and low scleral rigidity (4). Microcoria may also be present. Instrumentation within tight confines risks inadvertent iatrogenic injury to surrounding tissues. In our experience, the ultrashort instrument can be safely manoeuvred and does not bend easily despite its small gauge due to enhanced rigidity. Strategies to circumvent poor scleral rigidity and risk of AC collapse include smaller corneal incisions and the use of viscoelastic. Using the microincisional vitrectomy platforms, incision diameter can be as small as 0.4 mm with the 27G trocar/cannula system ensuring a better corneal wound integrity and AC stability compared to a 1.8 mm incision using the I/A probe. The versatility of the cutter allows single instrumentation for various steps of the surgery including anterior capsulotomy (for younger children with more elastic anterior capsule), lens aspiration, posterior capsulotomy, anterior vitrectomy without the need for repeated exchange of instruments. Possible theoretical advantages include reduced risk of inflammation and infection, and with less intraocular instrumentation and reduced surgical duration. Surgical duration is an important factor to consider against the backdrop of general anaesthesia exposure, especially amongst infants.

In infants and neonates, considerations for early surgery must be balanced with the systemic and ocular risks from early intervention (earlier than 4 weeks of age) from general anaesthesia and the increased glaucoma risk, respectively (12–18). Cataract surgery performed before the first month of life is associated with a fourfold increase of risk of secondary glaucoma (13). Mechanisms of secondary glaucoma include postoperative inflammation, structural change or anatomical destruction consequent upon surgery (13). In a 10-year follow-up study by Haargaard et al., 32% of eyes that underwent cataract surgery < 9 months of age developed glaucoma compared to only 4% of those ≥ 9 months of age (15). The IoLunder2 cohort study identified younger age ( ≤ 6 months) as the independent factor for early postoperative glaucoma in bilateral cataract cases and demonstrated a 2% risk reduction with each increasing week of age; whilst in unilateral cases, significant microphthalmia was the independent predictor for glaucoma (18). In the present cohort, 33% of those younger and 25% of those older than 6 months of age developed an elevated IOP postoperatively. Refractory IOP rise occurred in two cases having undergone surgery at age 2 months without primary IOL implantation. Long-term follow-up is outside the scope of this study, however. Primary IOL implantation for children younger than 2 years, with unilateral or bilateral cataracts, is generally avoided as they have not been found to confer better vision or protection against secondary glaucoma, conversely, they are associated with increased risk of early reoperation (19). In unilateral cases operated at ≤ 6 months of age, the Infant Aphakia Treatment Study (IATS) found glaucoma-related adverse events to be common and increased between age 1–5 years with young age being a risk factor that is not mitigated by primary IOL implant (20). Nevertheless, the topic of primary IOL implantation in infants remains controversial (7, 21). In developing nations and low-income populations, primary IOL implantation is generally preferred when the child meets the lower cut-off for AL and corneal diameter measurements recommended by IATS due to limited compliance with contact lenses or aphakic glasses (22, 23). Glaucoma is a major sight-threatening complication which must be discussed with parents and caregivers during pre-operative counselling as the development may occur decades after paediatric cataract surgery thus lifelong surveillance is recommended (15, 18).

Rapid visual axis opacification following cataract extraction is a common sequela, especially in young children up to the age of 7 years due to rapidly dividing lens epithelial cells. Strategies to minimise this include central posterior capsulotomy/capsulorhexis and anterior vitrectomy to break the scaffold for proliferating lens epithelial cells. Additional measures include capsular polish, optic capture, pars plicata posterior capsulorhexis, sutureless vitrectomy, sealed-capsule irrigation, and bag-in-the-lens IOL (24–27). Significant posterior capsule opacification may still develop and requires prompt intervention either with Nd:YAG capsulotomy or membranectomy to prevent amblyopia. In older children, the option to perform in-office Nd:YAG laser posterior capsulotomy with good outcomes obviates the need for additional operative measures (28). In the present cohort, 37% of those younger than 2 years of age and 25% of those older than 2 developed PCO.

In all but 2 secondary cataract cases, the causal relation could be easily established. These 2 cases had been diagnosed with LCA and had developed asymmetric cataract at 6 years of age. LCA is not commonly associated with childhood cataract. We hypothesise that the oculodigital reflex that these patients demonstrate might not only be a risk factor for corneal ectatic disease but could also affect the lens effectively making this a low-grade traumatic cataract. It is important to note that in many of the secondary cases with retinal disease cataract surgery wasn’t done with the primary goal of improving vision and/or preventing amblyopia but rather to be able to visualise the posterior segment during an in-office exam and/or was done during posterior segment surgery.

In our sub-cohort of syndromic (*n* = 4) and non-syndromic (*n* = 15) primary, bilateral, congenital cataract (37.3%), a monogenic molecular explanation was found in 5 patients (26.3%) using the cataract panel for genetic testing. This is a much lower than the rate found by Gillespie et al. who found a molecular explanation in 75% of bilateral congenital cataract cases (5). The lower-than-expected rate of molecularly explained cases in our cohort might (in part) be explained by the fact that it is solely composed of surgical cases and does not include cases of congenital cataract that did not require lens extraction.

Even though many patients with Down syndrome are referred to GUH for ophthalmic screening each year, only a single case had been diagnosed with cataract requiring lens extraction. The patients with Down syndrome in this cohort was diagnosed with cataract within the first year of life and was bilateral symmetrical. Haargaard et al. found that, at a rate of 1.4%, childhood cataracts are rare in Down syndrome with even less requiring surgery (29).

Unilateral congenital cataract is often associated with PFV which can be classified as anterior, posterior or combined. In the sub-cohort of unilateral congenital cataract (27.5%) are 3 cases of PFV. This makes for a rate of 21.4%. This rate is much lower than the number found in the loLunder2 cohort where 46% of cases had PFV (30). The present cohort had no cases of bilateral PFV, which is rare and has been associated with chromosomal abnormalities (30, 31).

One case in the present cohort had been diagnosed with the very rare Hallermann-Streiff-François syndrome with fewer than two-hundred cases reported in the literature. Its aetiology is unclear, and the disorder seems to occur sporadically. The majority (90%) of cases have ocular abnormalities of which bilateral congenital cataracts and microphthalmia are the most frequent (32, 33).

In children, accurate visual assessment can be challenging, often confounded by issues with inattention and limited cooperation particularly in those under the age of 4. The level of experience of the examiner in evaluating paediatric vision is of equal importance. The need for age-appropriate visual assessment tools from grating acuity for the pre-verbal children to recognition acuity using optotypes for older children makes trending or comparison of visual acuity pre-and post-intervention difficult. Although various methods have been developed and validated for the assessment of vision in infants and young children, it remains unclear whether these methods provide reliable visual assessment (2, 34). In this study, comparison between pre-and post-operative visual acuity demonstrated an improving trend despite the latter being taken prior to initiation of amblyopia treatment. Given the broad age range in the cohort with children at different stages of their visual rehabilitation at the time of data extraction, postoperative VA prior to initiation of amblyopia treatment was deemed most reflective of surgical impact.

The complexity of paediatric cataract management necessitates a uniform national care pathway. Early diagnosis and timely referral where surgery is required are only the first steps in maximising visual outcomes. Postoperative visual rehabilitation including rigorous amblyopia management, close monitoring during the early visual development phase and long-term surveillance for complications are of paramount importance.

This study has a relatively diverse patient cohort based on age and underlying aetiology of the cataract and introduces new instrumentation to the surgical armamentarium for the management of paediatric cataract. Limitations of this study include its retrospective nature with missing datapoints precluding in-depth analyses for certain domains such as pre-operative keratometry measurements and IOL prediction. The study does not have sufficient longitudinal follow-up data to determine long term surgical complications such as glaucoma and retinal detachment following cataract surgery.

## 5. Conclusion

This retrospective study describes a monocentric paediatric cataract cohort who underwent cataract surgery. In 13.7% of this cohort, novel or known suspect variants in cataract genes were identified. Many different instruments and techniques have been described and used in the context of paediatric lens extractions, each with its advantages and disadvantages. This study illustrates that an ultra-short 27G vitrectomy system can be used to perform paediatric lens extractions with good surgical outcomes. Further studies and comparative trials are needed to ascertain this further. Technological advances have revolutionised paediatric cataract surgery, but precise IOL calculation and timing of implantation remain a hurdle.

## Data availability statement

The data analysed in this study is subject to the following licenses/restrictions: due to large amount of data, patients would become identifiable. Requests to access these datasets should be directed to filip.vandenbroeck@ugent.be.

## Ethics statement

The studies involving human participants were reviewed and approved by Ethical Committee of Ghent University and Ghent University Hospital. Written informed consent from the participants’ legal guardian/next of kin was not required to participate in this study in accordance with the national legislation and the institutional requirements. Written informed consent was not obtained from the individual(s), nor the minor(s)’ legal guardian/next of kin, for the publication of any potentially identifiable images or data included in this article.

## Author contributions

HC and FB: data collection, analysis and most of the manuscript writing, and project coordination. AC: data collection and analysis and manuscript writing. SW and IJ: data contribution and case review and manuscript review. HV: molecular/genetics review and review of Table 2. IB and PD: data contribution and manuscript review. SN: data collection and manuscript review. EB: molecular/genetic testing. BL: project supervision and manuscript review. FN: surgeon/data source, project supervisor, and manuscript review. All authors contributed to the article and approved the submitted version.

## Funding

This study was supported by the Ghent University Special Research Fund (BOF15/GOA/011) to EB and by the Ghent University Hospital (NucleUZ to EB). EB (1802220N) and BL (1803821N) are Senior Clinical Investigators of the Fund for Research Flanders, Belgium (FWO).

## Conflict of interest

The authors declare that the research was conducted in the absence of any commercial or financial relationships that could be construed as a potential conflict of interest.

## Publisher’s note

All claims expressed in this article are solely those of the authors and do not necessarily represent those of their affiliated organizations, or those of the publisher, the editors and the reviewers. Any product that may be evaluated in this article, or claim that may be made by its manufacturer, is not guaranteed or endorsed by the publisher.
