# Supplementary material for: Paediatric cataract surgery with 27G vitrectomy instrumentation: the Ghent University Hospital Experience
Source: Front Med (Lausanne). 2023 Aug 3;10:1197984. doi: 10.3389/fmed.2023.1197984 (PMC10435324; doi:10.3389/fmed.2023.1197984)
Supplement: Supplementary file 2 [file Data_Sheet_1.docx]

Supplementary Table S1: Side-by-side comparison of D.O.R.C. 27G instrument dimensions. *With permission from D.O.R.C. International BV, Zuidland, the Netherlands.*

| **Instrument** | **Standard** | **Ultra Short** | **Difference** |
| --- | --- | --- | --- |
| Vitrectome working length | 28.5 mm | 21.0 mm | 26% |
| Trocar length | 4.0 mm | 3.0 mm | 25% |
| Inserter length | 10.3 mm | 7.65 mm | 25% |
| Illumination probe working length | 20 mm | 16 mm | 20% |
